# Short-chain fatty acids in diseases

**DOI:** 10.1186/s12964-023-01219-9

**Published:** 2023-08-18

**Authors:** Dan Zhang, Yong-Ping Jian, Yu-Ning Zhang, Yao Li, Li-Ting Gu, Hui-Hui Sun, Ming-Di Liu, Hong-Lan Zhou, Yi-Shu Wang, Zhi-Xiang Xu

**Affiliations:** 1 Key Laboratory of Pathobiology, Ministry of Education, Norman Bethune College of Medicine, Jilin University, Changchun, 130021 China; 2 School of Life Sciences, Henan University, Kaifeng, 475004 China; 3 Department of Urology, The First Hospital of Jilin University, Changchun, 130021 China

**Keywords:** Short-chain fatty acids, Gut microbiota, Inflammation, Metabolism, Immunity

## Abstract

Short-chain fatty acids (SCFAs) are the main metabolites produced by bacterial fermentation of dietary fibre in the gastrointestinal tract. The absorption of SCFAs is mediated by substrate transporters, such as monocarboxylate transporter 1 and sodium-coupled monocarboxylate transporter 1, which promote cellular metabolism. An increasing number of studies have implicated metabolites produced by microorganisms as crucial executors of diet-based microbial influence on the host. SCFAs are important fuels for intestinal epithelial cells (IECs) and represent a major carbon flux from the diet, that is decomposed by the gut microbiota. SCFAs play a vital role in multiple molecular biological processes, such as promoting the secretion of glucagon-like peptide-1 by IECs to inhibit the elevation of blood glucose, increasing the expression of G protein-coupled receptors such as GPR41 and GPR43, and inhibiting histone deacetylases, which participate in the regulation of the proliferation, differentiation, and function of IECs. SCFAs affect intestinal motility, barrier function, and host metabolism. Furthermore, SCFAs play important regulatory roles in local, intermediate, and peripheral metabolisms. Acetate, propionate, and butyrate are the major SCFAs, they are involved in the regulation of immunity, apoptosis, inflammation, and lipid metabolism. Herein, we review the diverse functional roles of this major class of bacterial metabolites and reflect on their ability to affect intestine, metabolic, and other diseases.

Video Abstract

**Supplementary Information:**

The online version contains supplementary material available at 10.1186/s12964-023-01219-9.

## Background

The human intestine contains a complex and diverse symbiotic microbial system that is mainly composed of bacteria, fungi, viruses, archaea, and protozoans [1]. Microbiota-derived metabolites are crucial mediators of host-microbial interactions. Recent studies have shown that the main metabolites of intestinal microbiota include short-chain fatty acids (SCFAs), secondary bile acids, trimethylamine, lipopolysaccharides (LPS), imidazopropionic acid, branched-chain amino acids, and indole and its derivatives, which affect metabolism, immunity, and tumour development [1, 2]. SCFAs are mainly produced by the intestinal microbiota from indigestible carbohydrates and host secretions via anaerobic fermentation and are one of the most important metabolite categories involved in the regulation of several biological functions [3, 4].

SCFAs regulate the structure of intestinal microbiota [5], enhance the function of the intestinal epithelial barrier [6], and are beneficial in delaying disease progression through a variety of ways, such as those in type 2 diabetes (T2D) [7], obesity [8], chronic kidney disease (CKD) [9], hypertension [9], inflammatory bowel disease (IBD) [10] and colorectal cancer (CRC) [7, 9–14]. Two signal transduction mechanisms mediate SCFA function; inhibition of histone deacetylase (HDAC) and activation of G protein-coupled receptors (GPCRs) [15]. HDAC-mediated epigenetic modifications play a vital role in gene expression [16]; thus, the inhibition of HDAC induced by SCFA affects the progression of diseases that include metabolic diseases [17], immune diseases [18], and cancer [19]. However, our understanding of SCFA-mediated inhibition remains incomplete [20]. GPCRs, particularly GPR43 (also known as free fatty acid receptor 2, FFAR2), GPR41 (FFAR3), and GPR109A (also known as hydroxycarboxylic acid receptor 2, HCAR2), have been identified as receptors for SCFAs. These GPCRs play important roles in the regulation of metabolism and inflammation [15, 21–24]. In this review, we summarise the classification, source, and role of SCFAs in diseases. The information is intended to provide a perspective for future studies of impact of SCFAs on diseases.

### Source and function of SCFAs

Saturated fatty acids with a chain length of 1 to 6 carbon atoms are defined as SCFAs [3, 25]. The most abundant SCFAs in the intestine are acetate, propionate, and butyrate [26]. SCFAs act on many cell types to regulate important biological processes, including host metabolism, intestinal function, and immunity [4, 27–31]. In the human colon and faeces, the molar ratio of acetate: propionate: butyrate is approximately 60:20:20 [21, 32]. Each SCFA is produced via bacterial fermentation. Therefore, the main reason for the different proportions of acetate, propionate, and butyrate was the catabolism of the different bacteria [26]. Cross-feeding with acetate- and propionate-producing bacteria, such as *Akkermansia muciniphila*, and butyrate-producing bacteria, such as *Faecalibacterium prausnitzii*, can improve the levels of intestinal SCFAs and have good preventive and therapeutic effects on inflammation and tumours [33]. This review focusses on the sources and functions of acetate, propionate, and butyrate.

### Acetate

**Fig. 1:**
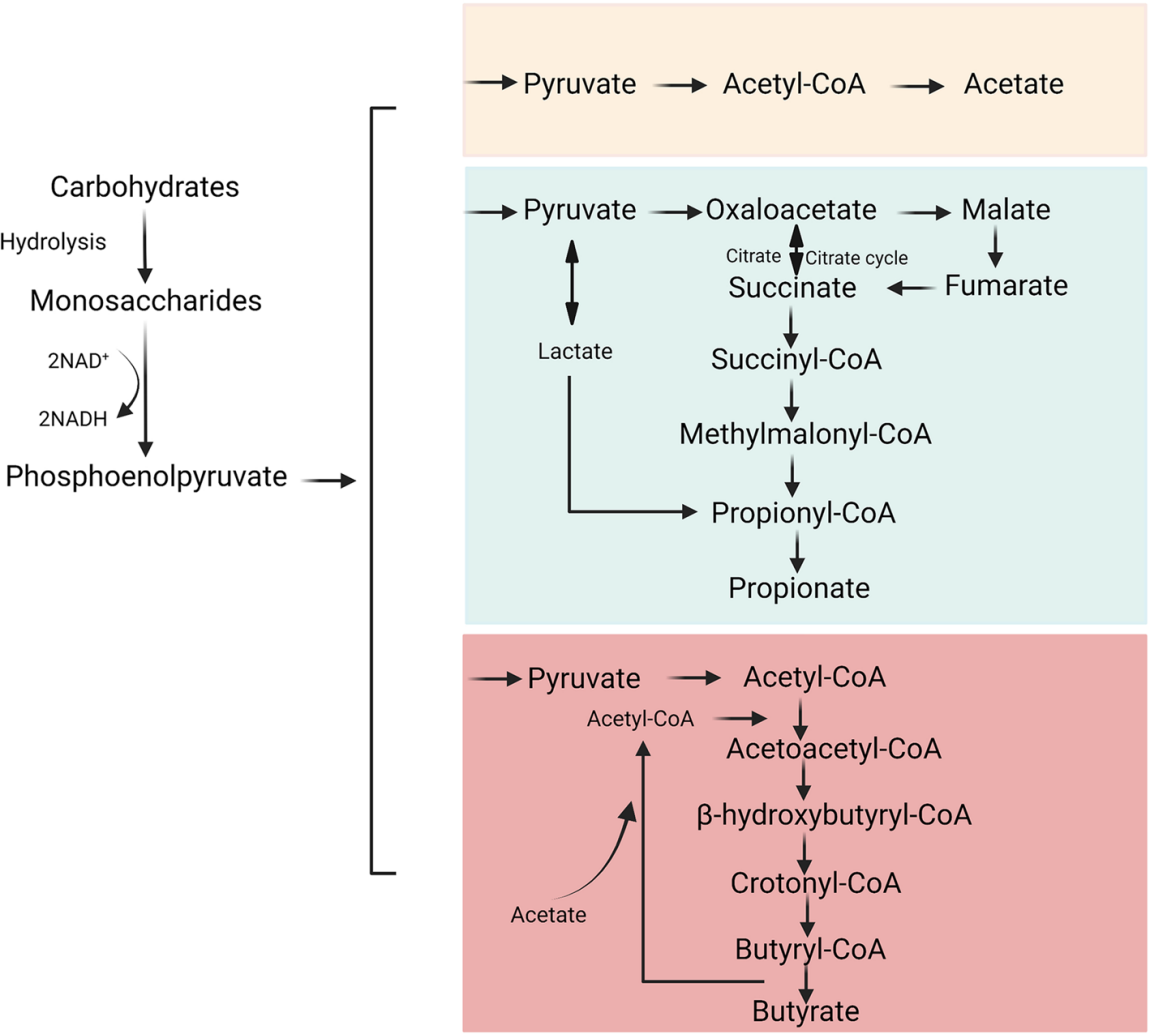
Schematic diagram of carbohydrate fermentation pathways producing acetate, propionate and butyrate

Acetate is mainly produced by anaerobic bacteria that digest dietary fibres in the animal colon. Several kinds of bacteria, such as *A. muciniphila* and *Bacteroides spp*. produce acetic acid through fermentation (Table 1) [21, 27, 34–36]. This process is mainly converted to acetate by acetyl-CoA produced by glycolysis, which is also enzymatically converted to butyrate by butyryl-CoA:acetyl-CoA transferase (Fig. 1) [21]. The highest concentration of this fermentation product is found in the proximal colon, where it is absorbed by intestinal epithelial cells (IECs) or transported to the blood through the intestinal epithelium and then quickly absorbed by the liver via the hepatic portal vein [15, 37]. In the blood, acetate exists as a free acid that is metabolised mainly in the liver [38], brain [39], heart [40], and muscles [41]. Acetate also has a wide range of effects on tissues and organs [42], is an important biofuel and nutrient source for tumour cells, and is involved in lipid synthesis [43]. In mice, fructose in the diet is converted to acetate by intestinal microbes, thereby providing acetyl-CoA for fat production [38]. In the brains of germ-free mice, acetate drives microglial maturation and regulates metabolic homeostasis [39]. Acetate maintains energy balance and metabolic homeostasis, resists oxidation and mitochondrial stress, affects immunity, and controls body weight and insulin sensitivity by affecting lipid metabolism and glucose homeostasis in animals and humans [38, 44–47]. Taken together, these data suggest that acetate is essential for lipid metabolism, weight control, neurological diseases, and tumour development of tumors. However, further studies are needed to explore the mechanisms by which acetate functions in these diseases and to determine the individual metabolic differences in the application of acetate.

**Table 1:** Sources of acetate, propionate, and butyrate

SCFAs	Origin	References
Acetate	*Akkermansia muciniphila*, *Bacteroides spp.*, *Bifidobacterium spp.*, *Prevotella spp.*, *Ruminococcus spp.*, *Escherichia coli*, *Blautia hydrogenotrophica*, *Clostridium spp.*, and *Streptococcus spp*.	[21, 27, 34–36]
Propionate	*Bacteroides uniformis*, *Bacteroides vulgatus*, *Prevotellacopr*, *Alistipes putredinis*, *Roseburia inulinivorans*, *Eubacterium hallii*, *Blautiaobeum*, *Coprococcuscatus*, *Dialisterinvisus Phascolarctobacterium succinatutens*, *Akkermansia muciniphila*, *Dialister spp.*, *Veillonella spp.*, *Megasphaera elsdenii*, *Coprococcuscatus*, *Bacteroides spp*., *Salmonella spp.*, *Roseburia inulinivorans*, and *Ruminococcus obeum*	[21, 34, 48–51]
Butyrate	*Clostridium* clusters IV and XIVa, *Faecalibacterium prausnitzii*, *Ruminococcus bromii*, *Lachnospiraceae*, *Eubacterium rectale, Roseburia spp.*, *Roseburia inulinivorans*, *Roseburia intestinalis*, *Eubacterium hallii*, *Anaerostipes hadrus*, *Anaerostipes spp.*, *Coprococcus eutactus*, *Coprococcus comes*, *Coprococcuscatus*, *Subdoligranulum variabile*, *Eubacterium biforme*, *Actinobacteria*, *Fusobacteria*, *Spirochaetes*, and *Thermotogae*	[34, 48, 50, 52–55]

### Propionate

Propionate is primarily derived from carbohydrate metabolism during glycolysis, mainly through the succinate pathway [48] (Fig. 1), as indicated by the widespread distribution of the methylmalonyl-CoA decarboxylase (mmdA) gene in Bacteroidetes and many Negativicutes [56]. *Bacteroides* and gram-negative bacteria, such as *A. muciniphila*, and *Roseburia inulinivorans,* are frequent human coliforms (Table 1) [21, 34, 48–51, 57].These microorganisms are involved in maintaining human health [34, 48, 56]. Zhang et al. described that propionate in chickens increased the secretion of glucagon-like peptide-1 (GLP-1) by IECs, inhibited hepatocyte adipogenesis, and reduced fat deposition in vitro [57]. Propionate also participates in liver gluconeogenesis in rats by acting on hydroxy-methylglutaryl-CoA reductase (3-hydroxy-3-methylglutaryl-CoA, HMG-CoA) to inhibit the synthesis of cholesterol in the liver [58, 59]. As a GPR41 agonist, propionate targets the enteric nervous system and exerts neuroprotective effects in mice with 6-hydroxydopamine-induced Parkinson’s disease (PD) [60]. Studies have suggested that T lymphocytes are sensitive to immunological challenges in the body, which is further supported by animal studies showing that propionate supplementation is either neutral or beneficial for host immune activity against bacteria and viruses [61]. In addition, propionate has a vascular protective effect in NMRI mice; it first acts on the immune system to inhibit the activity of helper T cells, and then on the cardiovascular system to reduce cardiovascular damage from hypertension [62, 63]. In C57BL/6 J mice, propionate inhibits intestinal cholesterol absorption, regulates dyslipidaemia, and reduces the area of aortic atherosclerosis [64]. These findings indicate crucial roles of propionate in lipid metabolism, the nervous system, and cardiovascular diseases. Although studies have shown that propionate is neutral or beneficial to the host by destroying bacteria and viruses, current knowledge is insufficient to explain how propionate supplementation affects immune responses to specific pathogens.

### Butyrate

Many commensal bacteria such as *Clostridium* clusters IV and XIVa, and *F. prausnitzii,* promote the synthesis of butyrate, which is then absorbed by human host IECs [14, 25, 65] (Table 1) [34, 48, 50, 52–55]. As an important metabolite off gut microbes, butyrate plays a critical role in the maintenance of energy supply in colon cells [27]. Butyrate is produced from carbohydrates via glycolysis via the combination of two acetyl-CoA molecules to form acetoacetyl-CoA, followed by a stepwise reduction to butyryl-CoA. The final step in the formation of butyrate from butyryl-CoA involves two different approaches; either the butyryl-CoA:acetate CoA-transferase route or the phospho-butyrate and butyrate kinase pathways (Fig. 1) [48]. Butyrate restores the epithelial barrier of the airway by inhibiting interleukin (IL)-6 production and by reducing IL-4 and zonula occludens protein 1 (ZO-1) expression in 16HBE human bronchial epithelial cells, which induces extracellular signal-regulated protein kinase 1/2 (ERK1/2) and c-Jun N-terminal kinase (JNK) phosphorylation [66]. Butyrate provides energy for normal IECs, whereas cancerous colon cells depend on glucose as their main energy source due to Warburg effect. Butyrate accumulates and acts as an HDAC inhibitor preventing the proliferation of HCT116 colon cancer cells [67]. Butyrate participates in maintaining the integrity of colonic mucosa in BALB/c mice, resisting colitis, and preventing the occurrence and development of cancer by regulating cell proliferation, apoptosis, and differentiation [27, 42, 68]. In intestinal diseases, butyrate enhances intestinal barrier function and suppresses intestinal inflammatory responses in mice by interacting with GPCRs and inhibiting HDACs [69]. These findings indicate that butyrate improves colonic inflammation and inhibits the occurrence and development of colon cancer, mainly by enhancing intestinal barrier integrity.

### Receptors for SCFAs

GPCRs are the largest family of receptors in mammals, comprising seven transmembrane domains. These receptors participate in the regulation of almost all cellular physiological functions in vivo [70]. GPCRs can bind to chemicals in the extracellular environment, such as hormones, neurotransmitters, chemokines, sugars, and lipids [71]. GPR41 and GPR43 are considered the most important SCFA receptors in the GPCR family [72]. A recent study demonstrated that SCFAs can also activate GPR109A. Unlike GPR41 and GPR43, GPR109A is activated by longer SCFAs, mainly C4 [73]. Different SCFAs receptors have different affinities for each other. In humans, the affinity ranking of GPR41 is C3 = C4 = C5 > C2 > C1, and that of GPR43 is C2 = C3 > C4 > C5 = C1 [71, 74]. GPCRs are reportedly associated with metabolic diseases [75], neurological diseases [76], inflammation [77], cardiovascular disease [78], and cancer [79]. Therefore, GPCRs have attracted attention as potential therapeutic targets for various of diseases.

### GPR41

GPR41 is a Gi/o-coupled receptor for SCFAs with ligands including acetate (C2), propionate (C3) and butyrate (C4) [80]. Recent findings have shown that SCFAs produced by microbial fermentation act as signalling molecules through receptors, such as GPR41 [81]. Thus, the gut microbiome plays a key role in host physiological and pathological processes via these receptors. Exogenous supplementation with SCFAs reduces liver fat content and improves liver metabolism by inhibiting the expression of lipid synthesis genes in GPR41^−/−^ mice [82]. The effect of propionate on allergic inflammation is dependent on GPR41 but not on GPR43. Activation of GPR41 by propionic acid treatment leads to changes in bone marrow haematopoietic function in mice, characterised by enhanced production of macrophages and dendritic cell precursors, followed by implantation of highly phagocytic dendritic cells in the lungs, which shape the immune environment of the lungs and influences the severity of allergic inflammation [83]. In addition, SCFAs promote IL-22 production by human CD4^+^T and innate lymphoid cells through GPR41, thereby protecting the intestinal tract from inflammation and maintaining intestinal homeostasis [18]. Therefore, GPR41, as an SCFA receptor, plays an important role in immune, inflammatory, metabolic, and other diseases, and is worthy of further exploration.

### GPR43

GPR43 is activated by acetate (C2), propionate (C3), and butyrate (C4) [80]. GPR43 is a Gi/o- and Gq double-coupled receptor expressed in intestinal endocrine cells, such as L cells [84]. GPR43 is also present in immune cells, such as monocytes, neutrophils, eosinophils, and regulatory T cells (Tregs) [85–87]. Levels of regenerating islet-derived protein 3 gamma (RegIIIγ) and β-defensin expression levels are decreased in IECs of GPR43^−/−^ mice. The oral administration of SCFAs in wild-type mice maintains intestinal homeostasis by activating the mammalian target of rapamycin (mTOR) and signal transducer and activator of transcription 3 (STAT3) pathways in IECs to produce antimicrobial peptides [88]. In T2D and diabetic nephropathy mouse models induced by high fat diet (HFD) and streptozolectin, GPR43 activation inhibits hyperglycaemia-induced oxidative stress and nuclear factor-κB (NF-κB) activation, enhances the interaction between β-arrestin-2 and I-κBα, and reduces kidney damage. Silencing of GPR43 by short interfering RNA inhibits this effect in mouse glomerular mesangial cells [89]. GPR43-deficient mice beccome obese under normal diet conditions, whereas mice specifically expressing GPR43 in adipose tissue remain slim even when fed a HFD. SCFA mediated GPR43 activation inhibits insulin signallings in adipose cells, thus inhibiting fat accumulation in adipose tissue and promoting lipid and glucose metabolism in other tissues. These findings implicate GPR43 as a sensor of excess dietary energy, thus controlling energy utilisation and maintaining metabolic balance [90]. GPR43 is expressed in adipose tissue, intestinal tract, and islet cells, and plays important roles in various physiological functions.

### GPR109A

GPR109A was originally identified as a nicotinic acid receptor that is activated by β-hydroxybutyrate and butyrate but not by acetate and propionate [91]. GPR109A is a Gi/o coupling protein expressed in colonic epithelial cells. The protein is downregulated in germ-free mice [92]. Butyrate alleviates osteolysis in mice by activating its receptor GPR109A, thereby inhibiting the activation of NLR family pyrin domain containing 3 (NLRP3) inflammasome induced by titanium particles [93]. Obese mice treated with butyrate displayed improved glucose metabolism and inhibited adipose tissue inflammation via GPR109A activation. Thus, targeting GPR109A to reduce metabolic and inflammatory dysfunctions is a potential new approach for treating obesity [94]. GPR109A activated by butyrate promotes the anti-inflammatory properties of colon macrophages and dendritic cells in C57BL/6 mice, induces the differentiation of Tregs and IL-10-producing T cells, and inhibits colon inflammation and carcinogenesis [95]. The collective findings demonstrate that GPR109A is expressed in many organs and cells and is beneficial for metabolism and immunity. The mechanism requires further study.

### SCFAs and diseases

**Fig. 2:**
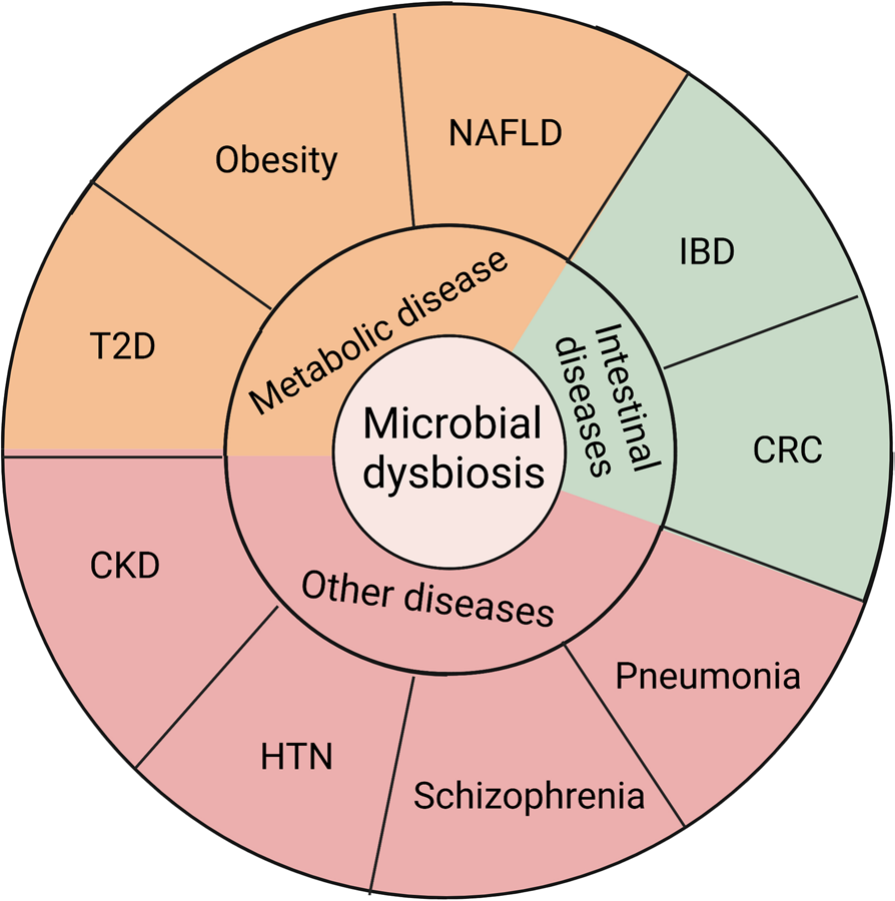
Impact of intestinal microbes on human diseases. T2D: type 2 diabetes; NAFLD: non-alcoholic fatty liver disease; IBD: inflammatory bowel disease; CRC: colorectal cancer; HTN: hypertension; and CKD: chronic kidney disease

SCFAs are closely associated with the occurrence and development of metabolic diseases, inflammation, and tumours (Fig. 2). An increasing number of studies have indicated that SCFAs act on membrane protein receptors GPCRs [96], and intracellular enzymes in IECs, or enter the circulatory system to regulate energy, glucose, and lipid metabolism [97]. SCFAs may function in three ways. The first is via interactions with GPR41, GPR43, and GPR109A, which are expressed in various organs that include the intestine, kidney, and heart [32]. The interactions are involved in the stimulation of phospholipase-Cβ, leading to the release of intracellular Ca^2+^ and activation of protein kinase C, in addition to cAMP accumulation and protein kinase A and ERK activation [98]. The second way is via transcriptional regulation and post-translational modifications. SCFAs, particularly butyrate, act as HDAC inhibitors [99, 100], promoting gene expression and regulating cell metabolism, differentiation, and proliferation [67]. In the third way, SCFAs enter cells through transporters such as the monocarboxylate transporter (MCT1) and sodium-coupled MCT (SMCT1) [3], which regulate cellular glucose metabolism, lipid metabolism, and immune function [21]. In this review, we summarise the mechanisms through which SCFAs ameliorate multiple diseases. The intent is to provide a perspective for future studies exploring the effects of SCFAs on diseases.

### Intestinal diseases

#### IBD

IBD, which includes ulcerative colitis and Crohn’s disease, is an idiopathic intestinal inflammatory disease of the ileum, rectum, and colon [101]. Both IBD subtypes are characterised by repeated cycles of epithelial damage, infiltration of inflammatory cells into the lamina propria, and failure of immune regulation to control the inflammatory response, resulting in recurrent cycles of chronic inflammatory remission and relapse in the gastrointestinal tract [102]. An imbalance in the intestinal microbiome is closely associated with IBD. SCFA-producing bacteria, such as* F. prausnitzii*, and *Roseburia intestinalis*, are significantly reduced in patients with IBD (Table 2) [21, 103–105]. Their reductions lead to impaired crosstalk between bacterial and immune cells. The concentrations of acetate and propionate are significantly reduced in the intestinal lumen of IBD patients [104]. SCFAs have important immunomodulatory effects due to their regulation of innate and adaptive immune cell generation, function, and trafficking, which play beneficial roles in IBD [102].

**Table 2:** Intestinal microbiota imbalance in various diseases

Disease	Microorganism (elevated)	Microorganism (reduced)	References
IBD	*Bifidobacterium*, *Staphylococcus*, *Enterococcus*, *Lactobacillus*, *Pseudomonas*, *Klebsiella*, and *Proteus genera*	Bacteroidetes, *Faecalibacterium prausnitzii*, *Roseburia intestinalis*, *Clostridium leptum*, and *Akkermansia muciniphila*, *Actinobacteria*, *Fusobacteria*, Proteobacteria, *Spirochaetes*, *Thermotogae*, *Butyricicoccus pullicaecorum,* and *Rhominis*	[21, 103–105]
CRC	*Bacteroides sp.*, *C. cocleatum, Collinsella spp*, *S. xylosus*, *P. excrementihominis*, *Muribaculum spp*, *A. equolifaciens*, *P. goldsteinii*, *F. aecalibaculum spp*, *B. faecis*, *Escherichia coli*, and *Bacteroides fragilis*	*Akkermansia muciniphila, B. pseudolongum*, *L. johnsonii*, *Olsenella spp*, *Prevotellasp, Colidextribacter spp*, *Bacillus spp*, *B. acidifaciens*, *L. reuteri*, and *E. faecalis*	[106, 107]
Obesity	Firmicutes	Bacteroides, and Bifidobacteria	[108–111]
T2D	Proteobacteria, *Lachnospiraceae*, *Ruminococcaceae*, and *Acinetobacter baumanii*	Bacteroides	[112–114]
NAFLD	*Anaerococcus*, *Ruminococcus*, *Peptoniphilus*, *Dorea*, *Bradyrhizobium*, *Propionibacterium acnes*, *Bateroides*, *Mucispirillum*, *Desulfovibrio*, *Anaerotruncus*, and *Desulfovibrionaceae*	Bacteroides*, Lactobacillus curvatus*, *L. plantarum*, *Bifidobacterium spp.*, *Rikenellaceae*, *Oscillospira*, *Prevotella*, *Bifidobacterium*	[115–118]
Pneumonia and respiratory diseases	*Coprobacillus*, *Clostridium ramosum*, and *C. hathewayi*	*Faecalibacterium prausnitzii*, *Bacteroides dorei*, *B. thetaiotaomicron*, *B. massiliensis*, and *B. ovatus*	[119, 120]
CKD	Firmicutes*,* Proteobacteria phyla*, Akkermansia muciniphila*, *Ruminococcus, Romboutsia*, and *Actinobacteria*	Bacteroides, *Faecalibacterium prausnitzii, Enterobacter*, *Enterococcus*, *Bifidobacterium*, *Roseburia*, *Clostridium*, *Coprococcu*,* and Lactobacilli*	[12, 106, 121, 122]
Hypertension	Bacteroides, *Klebsiella*, *Parabacteroides*, *Desulfovibrio*, *Lachnospiraceae*, *Ruminococcus*, *Actinobacteria*, and *Phascolarcto bacterium*	Proteobacteria, *Ruminococcaceae*, *Roseburia*, *Faecalibacterium spp.*, *Lactobacillus*, *Oscillibacter*, *Lachnospira*, *Prevotella*, and *Alistipes*	[13, 29, 123–125]
Neurologic disorders	*Streptococcus vestibularis*, *Akkermansia muciniphila*, *Bacteroides plebeius*, *Veillonellaparvula*, *Clostridium symbiosum*, *Eubacterium siraeum*, *Cronobacter sakazakii/turicensis*, *S. vestibularis*, *Alkaliphilus oremlandii*, *Enterococcus faecium*, *Bifidobacterium longum*, *Ruminococcaceae*, Bacteroides, *Coprococcussp.*, *Anaerococcus*, and *Lactobacillus fermentum*	Proteobacteria, *Faecalibacterium prausnitzii*, *Haemophilus spp*., *Sutterella spp.*, *Clostridium spp.*, *Gemmiger*, *Roseburia*, *Lachnospira*, and *Anaerostipes*	[126–128]

**Fig. 3:**
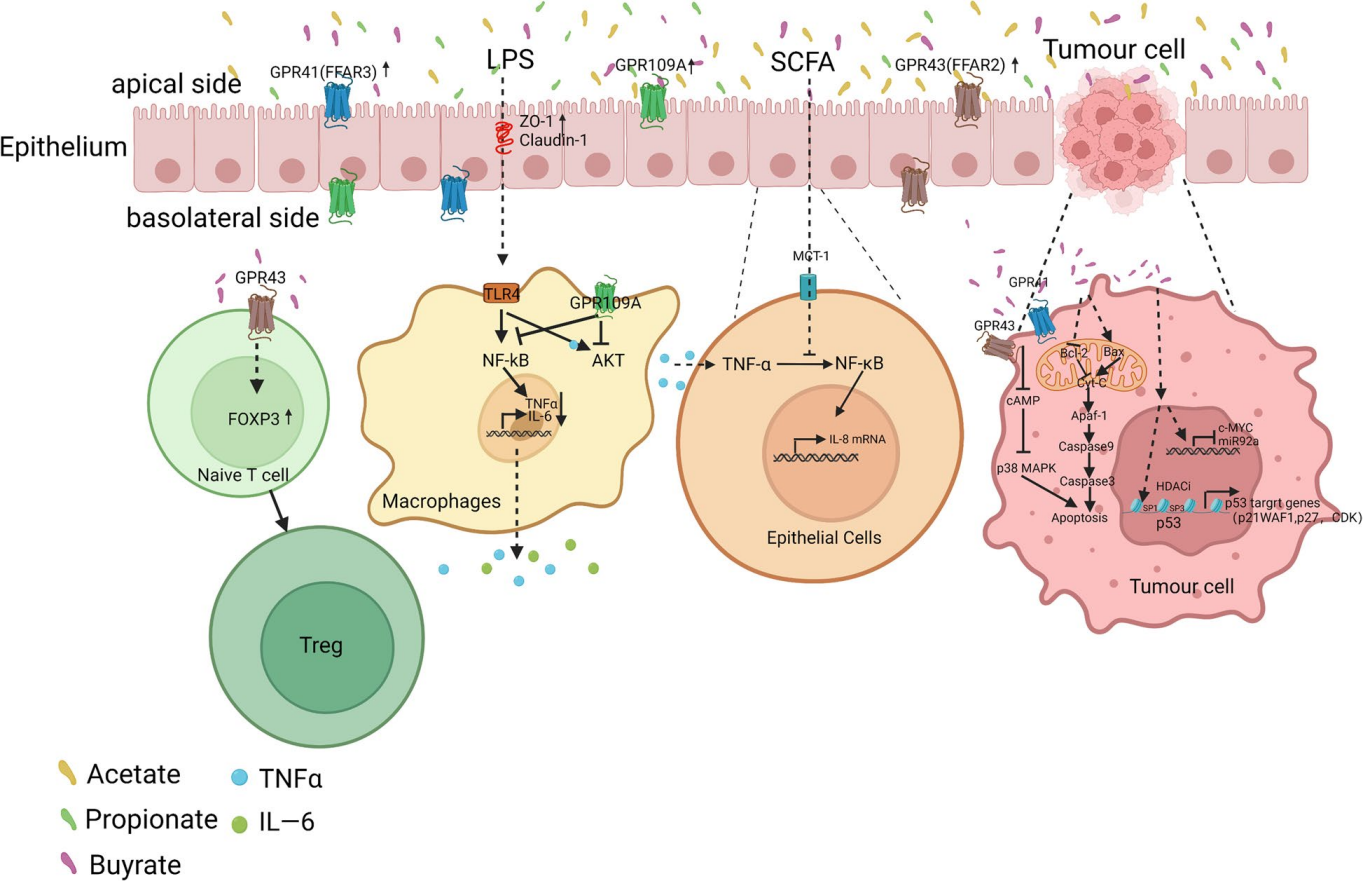
SCFAs alleviate IBD and CRC by increasing intestinal barrier function and reducing endotoxin levels in the blood. SCFAs increase the intestinal barrier function, reduce the entry of lipopolysaccharide (LPS) into the blood, induces naive T cells to differentiate into Tregs, inhibit the production of tumour necrosis factor-alpha (TNF-α) and interleukin (IL)-6 by intestinal macrophages, and inhibit the production of IL-8 by normal intestinal epithelial cells (IECs) to alleviate inflammation. Butyrate promotes CRC cell apoptosis by inhibiting C-AMP/P38-MAPK and increasing Bax/Bcl-2 ratio. Other abbreviations are: SCFA: short-chain fatty acid; GPCR: G protein-coupled receptor; FFAR: free fatty acid receptor; IBD: inflammatory bowel disease; CRC: colorectal cancer; AKT: protein kinase B; TLR4: Toll-like receptors 4; and HDACi: histone deacetylase inhibitor

Hung et al. showed that pretreatment of Caco-2 cells with acetate and propionate inhibited tumour necrosis factor-alpha (TNF-α)-induced inflammation by suppressing the activation of NF-κB p65 [129], spleen tyrosine kinase, and mitogen-activated protein kinase (MAPK) [130]. SCFAs, such as acetate, propionate, and butyrate, inhibit AKT and NF-κB by activating cell surface GPCRs. Inhibition of the NF-κB p65 signalling pathway reduces colon inflammation induced by 2,4,6-trinitrobenzene sulfonic acid (TNBS) in mice and ameliorates intestinal epithelial barrier dysfunction [131]. In a mouse model of colitis, butyrate treatment induced the differentiation of naïve T cells into Tregs by enhancing promoter histone H3 acetylation and a conserved noncoding sequence region in forkhead box protein 3 (Foxp3) [132]. In addition, SCFAs promote the expression of Foxp3 by activating GPR43 on T cells and induce the polarisation of intestinal T cells to Tregs in mice with colitis to inhibit the inflammatory response [133]. IBD is often accompanied by intestinal bleeding; Holtug et al. described that the concentration of SCFAs in the colons of IBD patients without intestinal bleeding was normal, while different concentrations of SCFAs were evident in the colons of IBD patients with bleeding [134]. In vitro co-culture of blood and faeces revealed that acetate and propionate levels were significantly reduced, whereas long-chain FAs, such as valerate, were significantly increased. The findings suggest that bleeding, as a risk factor in patients with IBD, contributes to the imbalance of SCFAs and accelerates the progression of IBD [134]. Concentrations of SCFAs can be decreased in IBD patients, and supplementation with SCFAs can send signals through GPCRs on the cell surface to activate signalling cascades that modulate immune function, thereby suppressing intestinal inflammation (Fig. 3). SCFAs are host-beneficial microbial products that can be supplemented through diet [21]. No significant adverse effects were observed in studies on SCFAs in IBD [104, 131, 135, 136].

### CRC

CRC has the third-highest incidence among gastrointestinal tumours [137]. The main types of CRCs are adenocarcinoma, mucinous adenocarcinoma, and undifferentiated carcinoma [138]. CRC is associated with HFD, stress, antibiotics, synthetic food dyes, monosodium glutamate, titanium dioxide, physical inactivity and/or sedentary behaviour, environmental factors, and other factors [139]. Among these, HFD is a key factor in CRC owing to the westernisation of the global diet, which involves high intake of red and processed meat, high fructose corn syrup, and unhealthy cooking methods [140]. Current research focuses on the role of different intestinal contents, such as fibres, vitamins, and metabolite imbalances in CRC [141]. The abundance of *Escherichia coli* and *Bacteroides fragilis* is reportedly higher in the intestines of patients with CRC, whereas the abundance of typical SCFA-producing bacteria, such as *A. muciniphila,* are significantly reduced (Table 2) [106, 107]. SCFAs are produced by catabolic fibre metabolism; butyrate is the main energy source for IECs. Butyrate inhibits the occurrence and development of colon cancer by regulating the expression of tumour suppressor genes and promoting apoptosis [142].

As an energy metabolite, butyrate promotes the proliferation of normal colon cells. Due to the Warburg effect, cancerous colonocytes rely on glucose as their primary energy source [67]. Therefore, butyrate accumulates in cancer cells, and high concentrations of butyrate acts as an inhibitor of HDAC and epigenetic inhibit the proliferation of cancer cells [143]. In HCT-116 cells, butyrate promotes pyruvate kinase isozyme 2 (PKM2) dephosphorylation and tetramerization to activate PKM2. This reprogrammes the metabolism of CRC cells, inhibits the Warburg effect, and promote energy metabolism [144]. Interestingly, in one study, the butyrate producer *Fusobacterium nucleatum* did not act as expected to inhibit colon cancer [145]. The authors reported that infection of HCT116 cells with *F. nucleatum* activated the Toll-like receptors 4 (TLR4)/ myeloid differentiation primary response 88 (MYD88)/NFκB signalling pathway, thereby increasing the expression of microRNA (miRNA) 21 (miR21) and promoting the malignant phenotype of colon cancer cells [145]. In patients with colon cancer, *F. nucleatum* activates the transcription of long noncoding RNA (lncRNA) ENO1 Intronic Transcript 1 (ENO1-IT1) by up-regulating the binding efficiency of transcription factor SP1 to the promoter region of lncRNA ENO1-IT1 and promoting glucose metabolism in CRC cells to induce cancer [146]. These findings suggest that the butyrate-producing bacterium *F. nucleatum* mainly promotes the development of colon cancer through other mechanisms, while the inhibitory effect of butyrate is weakened.

As an HDAC inhibitor, butyrate upregulates various signalling pathways, such as Janus kinase 2 (JAK2)/STAT, vascular endothelial growth factor, protein kinase C/WNT, and miRNA [147]. Butyrate promotes hyperacetylation of p53, which in turn increases the transcription and translation of p53 in RKO CRC cells [148]. Simultaneously, butyrate also promotes the acetylation of Sp1 and Sp3 in colon cancer HT-29 cells and further upregulates the expression of p53 targets (p21WAF1, p27, and cyclin-dependent kinases), induces cell cycle arrest, and promotes cancer cell apoptosis [149]. In addition, butyrate upregulates the expression of apoptosis-inducing factor 1, mitochondrial (AIFM1) to induce apoptosis in HT29 CRC cells [150]. In HCT-116 cells, butyrate inhibits the proto-oncogene c-Myc [151], thereby suppressing the transcription of miR-92a, promoting cell differentiation and apoptosis, and exerting anti-tumour effects in vivo [152, 153]. In a male BALB/c mouse model of colitis-associated CRC, tumours were induced by azoxymethane and dextran sodium sulfate. Combined administration of SCFAs can inhibit the expression of pro-inflammatory cytokines, including IL-6, IL-17, TNF-α, thereby preventing tumour development and reducing colon inflammation [154]. Therefore, butyrate induces CRC cell apoptosis by increasing the expression of apoptosis-inducing factors and tumour suppressor genes by acting as an HDAC inhibitor (Fig. 3). In the future, it will be interesting to test whether the addition of butyrate or administration of butyrate-producing bacteria foods, such as omega-3 polyunsaturated fatty acids, to the diet, will effectively prevent CRC and reduce the incidence of CRC.

### Metabolic diseases

Metabolic diseases include obesity, T2D, non-alcoholic fatty liver disease (NAFLD), thyroid disease, hyperuricemia, hyperlipidemia, and others. [155, 156]. The gut microbiota and SCFAs affect host metabolism, and dysbiosis is believed to be one of the main causes of common metabolic diseases in humans [2]. In mice, propionate and butyrate produced by the consumption of soluble dietary fibres activate intestinal gluconeogenesis (IGN) through a complementary mechanism [157]. IGN has beneficial effects on glucose and energy homeostasis, promotes metabolism, and regulates body weight and blood sugar levels. Butyrate activates IGN gene expression through a cAMP-dependent mechanism. Furthermore, as the substrate of IGN, propionate promotes IGN gene expression by activating the gut-brain neural circuit [157]. SCFAs promote IGN production to prevent metabolic diseases in mice [158]. Most studies investigating the effect of the gut microbiota and its metabolites on metabolic diseases have focussed on obesity, T2D, and NAFLD [156, 159], with few reports on thyroid diseases and hyperuricaemia. Therefore, the intestinal microbiota and its metabolites have relatively broad therapeutic prospects in the research of metabolic diseases, such as thyroid disease and hyperuricaemia. However, this review focusses on three widely studied metabolic diseases: obesity, T2D, and NAFLD.

### Obesity

Obesity is a global health concern. HFD and high-carbohydrate diets reduce the diversity and abundance of human intestinal microbes, leading to fat accumulation and obesity [160]. Studies have demonstrated that the ratio of Firmicutes/Bacteroidetes in the intestinal lumen of obese patients increases, whereas the abundance of the SCFA-producing bacteria *Bacteroides* and *Bifidobacteria* decreases (Table 2) [108–111, 161–163]. The host gains extra energy from SCFAs through the activity of gut microbiota [164, 165]. SCFAs are key factors involved in obesity resistance in both animals and humans [108].

**Fig. 4:**
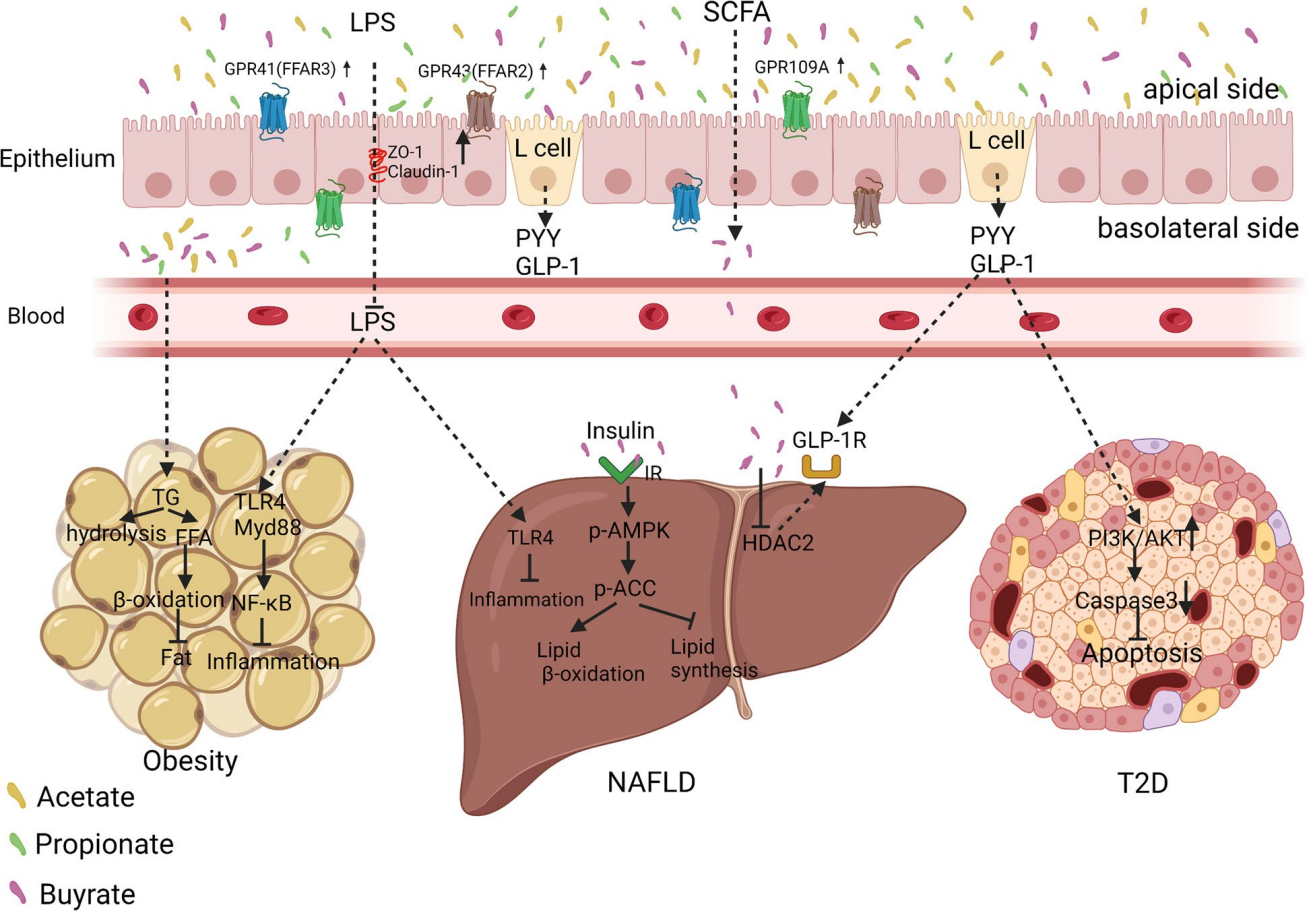
Intestinal microbiota metabolites acetate, propionate, and butyrate alleviates obesity, non-alcoholic fatty liver disease (NAFLD) and type 2 diabetes (T2D). Short-chain fatty acids (SCFAs) increase the intestinal barrier function by elevating the expression of zonula occludens-1 (ZO-1) and claudin-1 in IECs, preventing lipopolysaccharide (LPS) from entering the blood from the intestine, and further inhibiting adipose tissue inflammation. SCFAs promote the production of glucagon-like peptide-1 (GLP-1) in L cells; GLP-1 enters the liver and binds to the GLP-1 receptor on the surface of hepatocytes, promoting hepatic β-oxidation by activation of AMPK, thereby relieving fatty liver. GLP-1 activates PI3K/AKT signalling in pancreatic islets, inhibits apoptosis, and alleviates T2D. Other abbreviations are: GPCR: G protein-coupled receptors; FFAR: free fatty acid receptor; NF-κB: nuclear factor kappa-B; TG: triacylglycerol; TLR4: Toll-like receptor 4; p-ACC: phospho-acetyl-CoA carboxylase; p-AMPK: phospho-adenosine monophosphate-activated protein kinase; PYY: Peptide YY; and IECs: intestinal epithelial cells

In one study, 3T3-L1 mouse embryonic fibroblasts (preadipocytes) treated with acetate and propionate displayed expressions of GPR43, peroxisome proliferator-activated receptor-gamma 2 (PPAR-γ2), and leptin, which in turn promoted lipolysis metabolism [166]. In germ-free mice, inulin reportedly can restore the flora imbalance caused by HFD and promotes the production of SCFAs and IL-22, thereby preventing metabolic syndrome [167]. Lu et al. found that dietary supplementation with SCFAs, such as acetate, propionate, butyrate, or their mixtures, increased the expressions of GPR43 and GPR41 in adipose tissue, promoted the hydrolysis of triglycerides and promoted oxidation of free fatty acids in adipose tissue to produce brown fat, and reduced body weight in an HFD-fed mouse model [110]. In addition, supplementation of HFD mice with butyrate in increased the expression of PPAR-γ coactivator-1 alpha (PGC-1α), activated AMPK and p38, and enhanced insulin sensitivity [168]. *Ganoderma lucidum* is a medicinal mushroom that has been used to promote health, prolong life, and prevent diseases in Asian countries for more than 2000years [169]. The sporoderm-broken spores of *G. lucidum* (BSGL) increase the production of SCFAs and the expression of GPR43 in C57BL/6 J mice, promote the expression of ileal tight junction proteins and antibacterial peptides, improve endotoxaemia, and significantly reduce the upregulation of TLR4/Myd88/NF-κB signalling in adipose tissue induced by HFD (Fig. 4) [170]. Acute administration of inulin-propionate (which can be metabolised by the microbiota into propionate in the colon) to overweight adults significantly increases postprandial GLP-1 and peptide YY levels through the action of GPR43, promoting glucose decomposition and weight loss [108]. Reducing caloric intake and long-term propionate supplementation can significantly reduce weight gain [108]. Farup et al. tested the levels of SCFAs in the faeces of 90 patients undergoing bariatric surgery. The total SCFA levels were decreased 6 months after surgery, including those of acetate, propionate, butyrate, and branched-chain SCFAs [171]. The collective findings demonstrate the reduced SCFA content in the intestinal tract of obese patients, enhanced intestinal barrier function following SCFA supplementation, reduced endotoxaemia, promoted oxidative decomposition of free fatty acids, and reduced fat accumulation. However, few in-depth mechanistic studies have investigated the role of SCFAs in the treatment of obesity. Such studies are warranted.

### T2D

T2D is a metabolic disorder characterised by unbalanced blood sugar and lipid levels. Unbalanced gut microbiota is a factor in the rapid progress of T2D insulin resistance and account for approximately 90% of diabetes cases worldwide [172]. The abundance of Proteobacteria and the ratio of Firmicutes/Bacteroidetes are reportedly higher in T2D patients than in healthy subjects, whereas the abundance of SCFA-producing Bacteroides is reduced (Table 2) [112–114]. Levels of SCFAs, bile acids, and lipids in T2D patients are significantly dysregulated [112]. An increasing number of studies have shown that SCFAs play important roles in the restoration of insulin sensitivity [11].

Acetate and butyrate activate GPR43 and GPR41 on the surface of rat intestinal cells, promoting the secretion of insulin, GLP-1, and peptide YY, thereby regulating blood lipid energy metabolism and reducing peripheral blood glucose levels [173]. A human genome-driven increase in gut butyrate production is associated with improved insulin response after an oral glucose tolerance test, whereas abnormalities in the production or absorption of propionate are causally related to an increased risk of T2D [174]. Probiotics increase the activities of GPR43/41, proglucagon, and proconvertase 1/3, enhance insulin secretion through GLP-1 secretion triggered by glucose, and activate the phosphoinositide 3-kinase (PI3K)/AKT pathway to inhibit pancreatic cell apoptosis [174]. The findings show that SCFAs can reduce peripheral blood glucose levels and improve insulin resistance by increasing the levels of GPCRs and promoting the secretion of insulin, GLP-1, and peptide YY (Fig. 4). However, suitable doses and acceptable exposure times for SCFA treatment in T2D are undefined. In addition, whether there are time- and dose-dependent effects for SCFA treatment remains uncharacterized.

### NAFLD

The gut-liver axis refers to the bidirectional relationship between the gut and its microbiota and the liver, resulting from the integration of signals generated by dietary, genetic, and environmental factors [175]. This reciprocal interaction is established by the portal vein, which enables the transport of gut-derived products directly to the liver and the liver feedback route for bile secretion into the intestine [176]. The abundance of the SCFA-producing bacteria Bacteroides, *Lactobacillus curvatus*, and *L. plantarum* in the gut of NAFLD patients is significantly decreased (Table 2) [115–118]. Growing evidence supports the pathogenic role of microbe-derived metabolites, such as trimethylamine, secondary bile acids, SCFAs, and ethanol, in the pathogenesis of NAFLD [175]. Acetate provides a substrate for liver fat synthesis to promote fat production, whereas propionate can alter liver metabolism and reduce lipid storage, thus playing an important role in patients with NAFLD [177].

Expression of the GLP-1 receptor in the liver of NAFLD patients is significantly downregulated, and butyrate supplementation enhances the expression of the GLP-1 receptor in the livers of mice with NAFLD by inhibiting HDAC-2, which in turn promotes energy metabolism and inhibits lipid accumulation [178]. It also enhances insulin sensitivity, activates AMPK to promote the expression of fatty acid oxidation genes in hepatocytes, and reduces fat deposition in NFLAD mice [179]. In an HFD-induced NAFLD mouse model, dietary supplementation with sodium butyrate reportedly increased the abundance of *Christensenellaceae*, *Blautia,* and *Lactobacillus* in the intestine, forming a beneficial positive feedback cycle by producing more butyric acid [180, 181]. In another study, butyrate attenuated NAFLD-induced intestinal mucosal injury by increasing the expression of ZO-1 in the intestinal tract of mice, thereby preventing the migration of enterotoxins to the liver and inhibiting liver inflammation [182]. In humans, acute administration of inulin-propionate (which can be metabolised by the microbiota into propionate in the colon) significantly increases postprandial GLP-1 and peptide YY levels and decreases hepatic lipid deposition through the action of GPR43 [108]. Taken together, the findings demonstrate that SCFAs can promote liver energy metabolism to reduce fat deposition, enhance intestinal barrier function, and reduce ectopic toxins in the liver. These actions have protected against the development of NFLAD (Fig. 4).

### Other diseases

#### Pneumonia and respiratory diseases

Pneumonia is an infectious inflammation of the alveoli, airways, and lung interstitium. It is primarily caused by infections with bacteria, viruses, or other pathogens [183]. Normal upper respiratory tract and intestinal microbiota prevent pneumonia by preventing the colonisation of potentially pathogenic bacteria and regulating the immune response [184]. Coronavirus disease 2019 (COVID-19), caused by severe acute respiratory syndrome coronavirus 2 (SARS-CoV-2), primarily infects the respiratory system and affects other organs, such as the gastrointestinal tract [185]. Recent studies have reported altered gut microbiota in SARS-CoV-2 infections, characterised by the depletion of probiotics (butyrate-producing) [119, 186], such as several genera of the family *Ruminococcaceae* and *Lachnospiraceae* [187]. Gut microbiota are also disturbed in patients with other forms of pneumonia and respiratory diseases (Table 2) [188]. Mice fed a high-fibre diet have increased circulating SCFA levels, which protect the lungs from allergic inflammation, whereas low SCFA levels are associated with increased allergic airway disease [83].

**Fig. 5:**
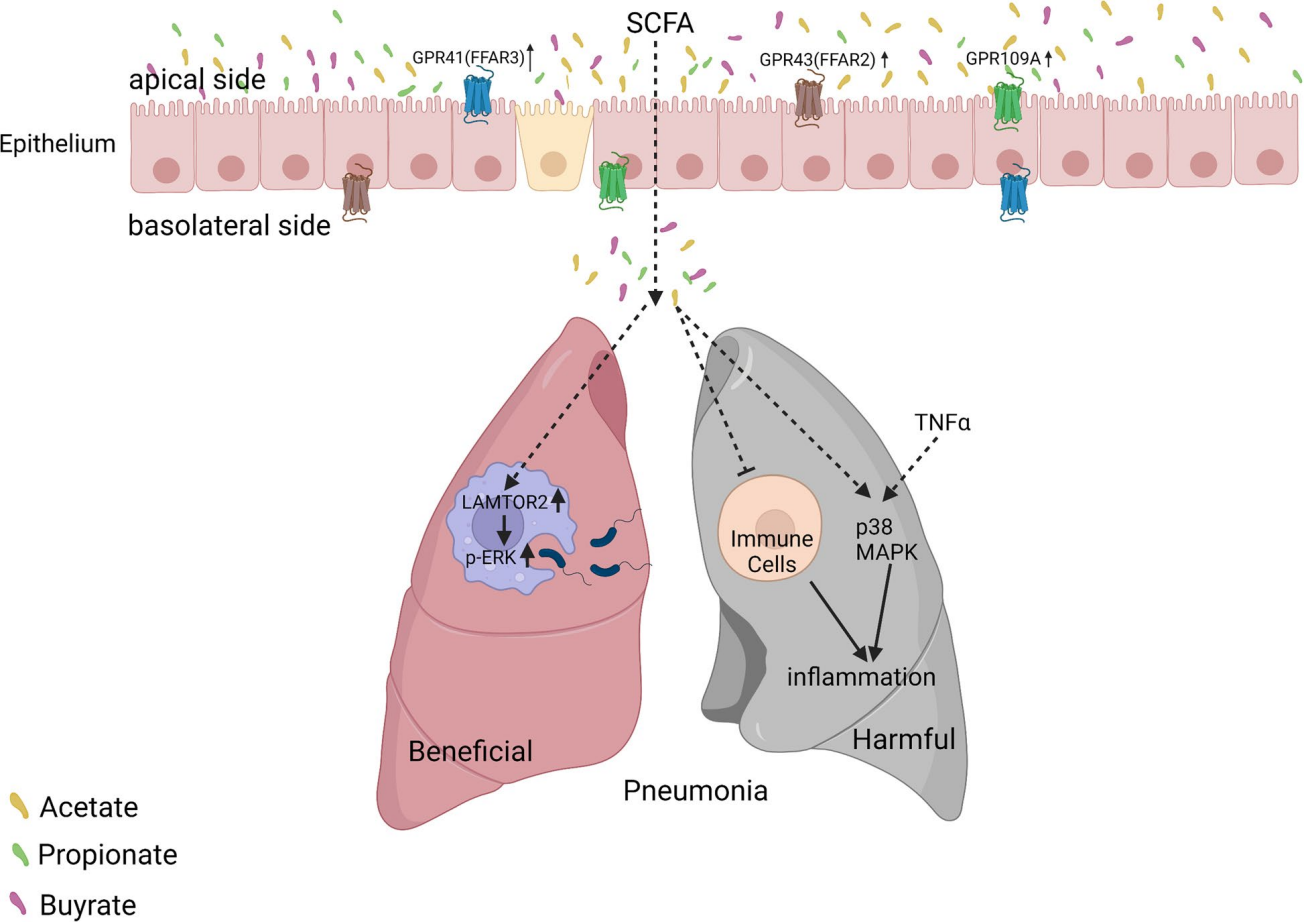
Beneficial and detrimental effects of SCFAs on pneumonia and other respiratory diseases. Short-chain fatty acids (SCFAs) promote the phagocytosis of Klebsiella. pneumoniae by pulmonary macrophages to clear lung bacteria. SCFAs can also inhibit lung immune cell function and promote inflammation. Other abbreviations are: GPCR: G protein-coupled receptors; FFAR: free fatty acid receptor; p-ERK: phosphorylated extracellular signal-regulated kinase; TNF-α: tumour necrosis factor-alpha; MAPK: mitogen-activated protein kinase

The role of SCFAs in pneumonia remains unclear. Propionate and acetate are important contributors to the gut-lung axis and may influence the immune response of piglets [189]. Propionate reshapes the mouse lung immune environment via GPR41 and reduces the severity of lung inflammation [83]. Furthermore, acetate and GPR43 increase bacterial clearance in mouse lung macrophages by upregulating late endosomal/lysosomal adaptor, MAPK, and mTOR activator 2 (LAMTOR2), which was further identified as an antibacterial effector and shown to facilitate phagosome-lysosome fusion and ERK phosphorylation [190]. However, not all studies have agreed on the anti-inflammatory effects of SCFAs. Rutting et al. investigated whether SCFAs inhibited TNFα-induced inflammation in primary human lung fibroblasts and airway smooth muscle cells in vitro and found that the combination of propionate and TNFα further promotes the phosphorylation of p38 MAPK and enhances inflammation [191]. Mammalian enteral malnutrition can aggravate allergic lung inflammation via T cell-and DC-dependent mechanisms that are inhibited by SCFAs [192]. In humans, SCFAs inhibit alveolar macrophage polymorphonuclear leukocyte phagocytosis by opsonised *Staphylococcus* and may cause anaerobic infections (Fig. 5) [193]. The role of SCFAs in pneumonia and respiratory diseases remains unclear. Whether SCFAs play a role in pneumonia and respiratory diseases depends on the patient’s nutritional status. Further research is required to clarify the role of SCFAs in respiratory diseases.

### CKD

In CKD, impaired renal function leads to the accumulation of uraemic toxins in the intestine, causing changes in bacterial composition and faecal metabolites [194]. Altered metabolites undergo positive feedback, which allows endotoxins to translocate into the blood, thereby enhancing local kidney inflammation, exacerbating kidney damage, and affecting CKD prognosis [9]. In patients with end-stage renal disease, the abundance of *Brachybacterium* and *Catenibacterium* increases in the colon, whereas those of *Lactobacillaceae* and *Prevotellaceae* decrease [195]. The administration of dietary fibre to mice with nephropathy increased the abundance of the SCFA-producing bacteria *Prevotella* and *Bifidobacterium* (Table 2) [12, 106, 121, 122]. Complex interactions exist among the brain, intestines, microbiota, and kidneys in patients with CKD and hypertension [154]. The pathogenesis of these diseases can be explained by the brain-gut-kidney axis [9]. Increased concentrations of SCFAs in faeces and blood circulation are associated with decreased levels of inflammatory cytokines and chemokines in the kidney via GPR43 or GPR109A [196].

**Fig. 6:**
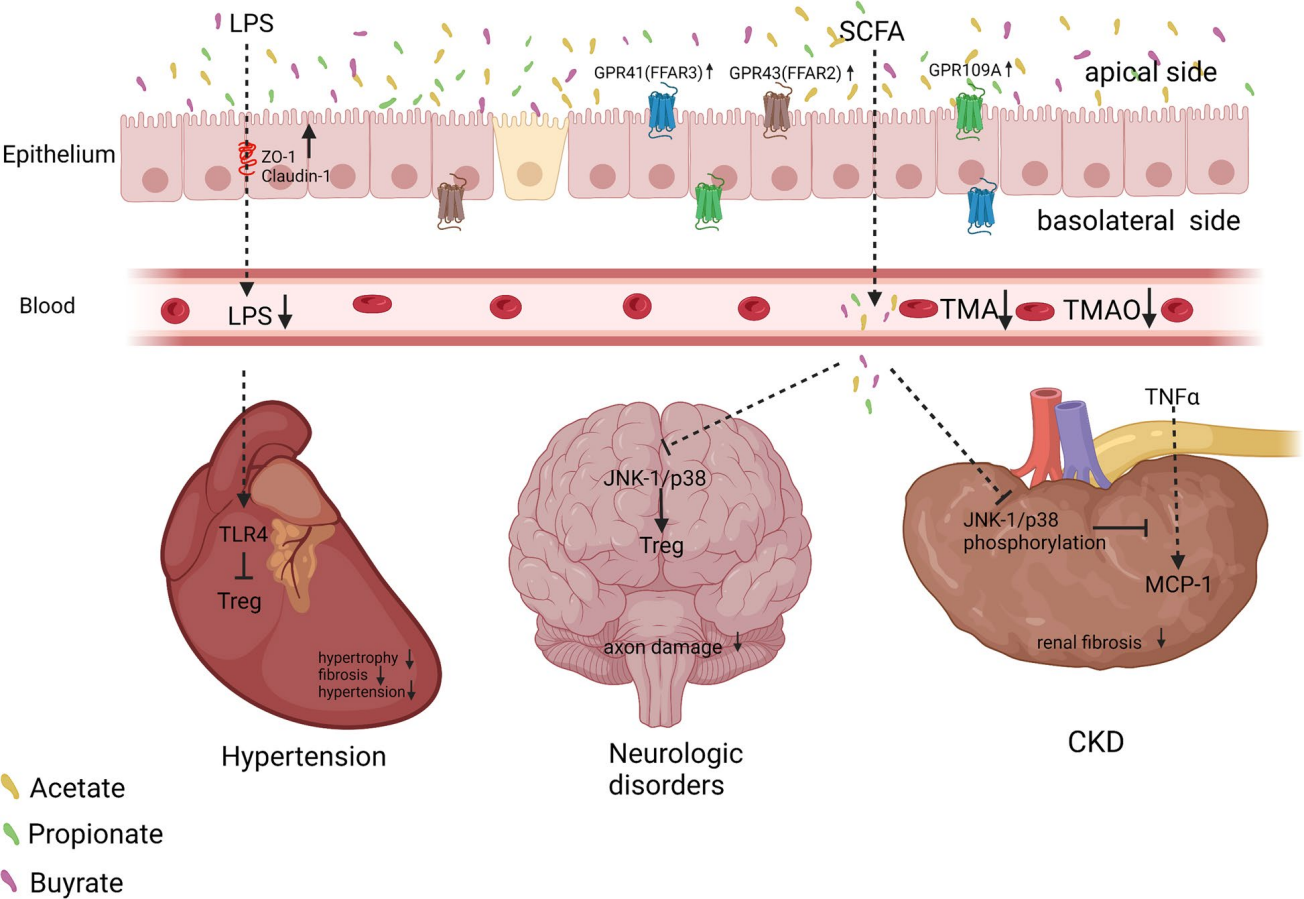
Short-chain fatty acids (SCFAs) alleviate hypertension, neurologic disorders, and chronic kidney disease (CKD) by modulating immunity. SCFAs alleviates hypertension by reducing lipopolysaccharide (LPS) entry into the blood and inhibiting cardiac Treg cells. SCFAs can also alleviate neurological diseases by reducing axonal damage via the inhibition of the c-Jun N-terminal kinase 1 (JNK-1)/p-38 pathway. In addition, SCFAs alleviate renal fibrosis by inhibiting the phosphorylation of JNK-1/p-38 pathway. Other abbreviations are: GPCR: G protein-coupled receptors; FFAR: free fatty acid receptor; and MCP-1: monocyte chemotactic protein-1

SCFAs, especially propionate, attenuate the expression of monocyte chemotactic protein-1 stimulated by TNF-α by inhibiting the phosphorylation of p38 and JNK in human renal cortical epithelial cells, thereby inhibiting renal inflammation and fibrosis [197]. Clinical studies have shown that butyrate levels in healthy volunteers are three times higher than those in CKD patients [12]. Administration of butyrate to rats with nephropathy improves renal fibrosis, reduces trimethylamine and trimethylamine N-oxide in the serum and faeces, and improves CKD progression in rats [12]. Diabetic mice given a high-fibre diet showed improved intestinal microecology, increased intestinal and systemic SCFAs, and inhibited kidney injury caused by GPR43 and GPR109A [121, 198]. These findings indicate that an imbalance in the intestinal microbiota of patients with CKD affects their SCFA metabolites, and the reduction of propionate and butyrate enhances the progression of CKD (Fig. 6). Therefore, supplementing SCFAs directly or modulating the gut microbiota that favours the production of SCFAs through dietary fibre or nutritional therapy may have a positive impact on the management of chronic renal failure.

### Hypertension

Hypertension is the most common chronic disease and the most important risk factor for cardiovascular and cerebrovascular diseases [199]. The composition of the gut microbiome is dysregulated in hypertensive patients, while SCFA-producing bacteria, such as *Roseburia spp*. and *Faecalibacterium prausnitzii*, are decreased (Table 2) [13, 29, 123–125].

Oral gavage application of acetate and butyrate in hypertensive rats inhibits the vascular LPS/TLR4 pathway, increased the infiltration of Treg cells into the vascular system, and reduced the number of Firmicutes in the ratio of Firmicutes/Bacteroides [200]. In a mouse model of angiotensin II-induced hypertension, mice administered a propionate gavage showed significantly reduced cardiac hypertrophy, fibrosis, vascular dysfunction, and hypertension [62]. Studies have shown that the cardioprotective effect of SCFAs in C57BL/6 J mice is mediated by the SCFA cognate receptor GPR43/GPR109A, which modulates L-3,4-dihydroxyphenylalanine levels and the abundance of Tregs regulated by DNA methylation [201]. Supplementation with SCFA in mice prevented dietary fibre deficiency-induced upregulation of calmodulin-dependent protein kinase II and NLRP3 inflammasome activation in atrial tissue [202]. Compared to individuals with normal blood pressure, faeces of hypertensive individuals display higher levels of acetate, butyrate, and propionate [203], whereas their levels in the plasma are lower [124, 204, 205]. Thus, SCFA-producing bacteria in the intestines of hypertensive patients and the content of SCFAs in the plasma are reduced. Therefore, it is possible that the absorption of SCFAs in hypertensive patients is significantly reduced and that SCFAs are excreted in the faeces. These findings have shown that the levels of SCFAs in the plasma of hypertensive patients are low, and supplementation with SCFAs improves immunity and suppresses inflammatory responses in patients, thereby relieving symptoms such as cardiac hypertrophy and fibrosis (Fig. 6). However, most studies testing the effects of SCFAs on hypertension have focussed on phenotypic descriptions, and their specific molecular mechanisms remain unclear. Further research is needed to elucidate the potential effects and underlying mechanisms of SCFAs in hypertension, which will provide fundamental information for new approaches for the prevention and treatment of hypertension.

### Neurologic disorders

The gut microbiota participates in the pathogenesis of neurological disorders through the gut-brain axis [206]. Compared to healthy volunteers, patients with neurological disorders, such as schizophrenia, PD, Alzheimer's disease, and autoimmune encephalomyelitis, have a significant imbalance in multiple characteristic bacteria in the gut, such as a significantly reduced abundance of the butyrate-producing bacterium *F. prausnitzii* (Table 2) [126–128, 207, 208]. Increased serum butyrate levels are associated with favourable treatment responses in drug-naïve first-episode schizophrenia patients [209]. SCFAs in the faeces of PD patients are reduced, and intestinal inflammation occurs, indicating that SCFAs may be candidate molecules and pathways for the pathogenesis of PD [210]. Dietary SCFAs help expand gut Tregs to modulate autoimmune responses by inhibiting the JNK1 and p38 pathways, thereby reducing axonal damage in patients with autoimmune encephalomyelitis [208]. The abundance of *Clostridium spp*. in the gut of patients with schizophrenia is significantly reduced, resulting in reduced production of SCFAs and aggravation of the disease [127]. Transplanting the faecal microbiota of patients with schizophrenia into antibiotic-treated mice can cause abnormal behaviours in the recipient animals, such as hyperactive psychomotor function and impaired learning and memory [211]. In another study, administration of a mixture of acetate, propionate, and butyrate to mice following a 3-week social defeat and overcrowding procedure alleviated the heightened stress-responsiveness and stress-induced increases in intestinal permeability while also decreasing anxiety-like behaviour in the open field test and decreasing depressive-like behaviour in the forced swim test [212]. To date, the roles of SCFAs in dietary intake and immune and metabolic outcomes have not been systematically studied in neurological disorders [213]. It remains unclear whether a high-fibre diet can ameliorate these diseases by increasing SCFA production. Whether SCFAs play a direct or indirect role in neurological disorders should be studied in detail (Fig. 6).

### Therapeutic relevance

#### Faecal microbiota transplantation (FMT)

FMT is a treatment method that transfers a faecal suspension obtained from a healthy donor to the patient's digestive tract to restore the normal microbial composition and function of the intestinal tract [214, 215]. FMT could have applications in the treatment of many diseases, such as ulcerative colitis, irritable bowel syndrome, asthma, PD [214, 216–219]. The results of FMT depend on the donor, and the use of super donors with normal organisms and favourable specific bacterial characteristics is critical for successful treatment [216]. In several studies, after FMT administration in patients with IBD, the relative abundances of *Eubacterium hallii* and *Odoribacter genera* increased, but the relative abundances of Bacteroides, *Helicobacter*, and *Clostridia* decreased [220, 221]. *E. hallii* and *Odoribacter* are the main bacteria that produce SCFAs and increase their concentration in the colon [222, 223]. SCFAs inhibit inflammation in mice by interacting with GPR43 to improve inflammatory diseases, such as colitis, arthritis, and asthma [224]. In addition, SCFAs in mice modulate B-cell differentiation through the GPR43 receptor and relieve rheumatoid arthritis [225]. FMT administration increases the concentration of SCFAs in the colon [209], and the NF-κB pathway is regulated to inhibit inflammation [214]. At present, frozen stool processing promotes the clinical application of FMT, making it possible to establish an FMT library [216, 226]. However, the specific bacterial composition of FMTs and the mechanisms underlying of FMT treatment remain unclear. Given the apparent effectiveness of this treatment strategy, further research is required to elucidate the precise underlying mechanisms.

### Dietary intervention

Dietary composition has the most significant effect on gut microbes [227, 228]. Different types of diets can change the composition of microorganisms, increase the ratio of harmful bacteria to their metabolites, and induce chronic metabolic diseases, such as obesity, and T2D [229, 230]. Healthy eating habits, which include consuming plenty of fresh fruits, vegetables, fish, extra virgin olive oil, and whole grains, can effectively prevent these diseases, whereas refined and processed foods such as sweet, fried foods, processed meats, and refined grains can increase the risk of illness [230, 231]. Mice fed a high-fibre diet showed increased levels of SCFAs and were protected against allergic inflammation in the lungs, whereasthose fed a low-fibre diet showed decreased levels of SCFAs and increased allergic airway disease [83]. Dietary fibres alter intestinal SCFA levels, maintain mucosal homeostasis and intestinal epithelial integrity, promote the growth of Tregs, and inhibit the expression of inflammatory cytokines to prevent and/or ameliorate diseases [232]. A low-calorie, low-protein, low-carbohydrate HFD was adopted as a fast-mimicking diet; this diet may promote cell regeneration by reducing the activity of protein kinase A and mTOR, inducing the expression of Sox2 and Ngn3, and restoring insulin generation, secretion, and glucose homeostasis in T2D mouse models and T1D patients [233]. Eating habits, that regulate physical health are more feasible and have certain advantages over drugs, surgical, and healthcare products.

### Prebiotic/probiotic applications

In recent years, research on prebiotics and probiotics has attracted considerable attention [234]. Their mechanisms of action are complex and diverse and are usually strain- and compound-specific [235]. Probiotics can change the microenvironment of the gastrointestinal tract, compete with pathogenic bacteria for nutrients, and inhibit the growth of pathogenic bacteria by producing strain-specific antimicrobial compounds [236, 237].

In the human body, probiotic effector molecules can directly interact with receptors in the intestinal epithelium, enteroendocrine cells, immune cells, and vagus nerve afferent fibres to enhance the local effects on the intestinal tract, such as the integrity of the intestinal barrier, inflammation, immunity, and the endocrine and enteric nervous systems [236]. In addition, probiotics-based interventions promote an increase in SCFA levels, insulin sensitivity, fat decomposition, and body metabolism [238]. SCFAs are involved in regulating human emotions and cognitive abilities and affect mental function through the gut-brain axis [3]. Changes in microbial composition and metabolite concentration caused by administration of prebiotics affect host epithelial, immune, neurological, and endocrine signals and thus mediate health benefits that include improved intestinal function, immune response, glucose and lipid metabolism, bone health, appetite, and regulation of satiety [239, 240]. Therefore, prebiotics and probiotics provide health benefits to the host and reduce side effects when applied in clinical settings and are expected to become effective treatments for many diseases.

## Conclusion

SCFAs are important metabolites produced by gut microbes. As the second-largest genome and ninth-largest system in the human body, the intestinal microbiota is critical for maintaining health. Bacterial imbalances and their metabolites lead to various diseases, including obesity, T2D, NAFLD, CKD, IBD, CRC, pneumonia, and schizophrenia. SCFAs affect the occurrence and development of various diseases in several ways. Among the diseases covered in this review, SCFAs mainly exert their effects by enhancing intestinal barrier function, inhibiting the inflammatory response, promoting apoptosis, increasing the expression of GPCRs, affecting histone acetylation, and regulating immunity. Currently, the mechanisms by which SCFAs function in various diseases are not fully understood. In the present review, we clarify the mechanisms of action SCFAs in various diseases. The therapeutic effects of faecal bacterial transplantation, dietary intervention, and probiotic/prebiotic supplementation on diseases through the regulation of microbial metabolites are described.

## Data Availability

Not applicable.

## Abbreviations

BSGL: Broken spores of Ganoderma lucidum
CKD: Chronic kidney disease
COVID-19: Coronavirus disease 2019
CRC: Colorectal cancer
ERK: Extracellular signal-regulated kinase
FMT: Fecal microbiota transplantation
FOXP3: Forkhead box protein 3,
GLP-1: Glucagon-like peptide-1
GPCRs: G protein-coupled receptors
HCAR2: Hydroxycarboxylic acid receptor 2
HDAC: Histone deacetylase
HFD: High-fat diet
HMG-CoA: 3-Hydroxy-3-methylglutaryl-CoA
IBD: Inflammatory bowel disease
IECs: Intestinal epithelial cells
IGN: Intestinal gluconeogenesis
IL: Interleukin
LAMTOR2: Late endosomal/lysosomal adaptor, MAPK, and mTOR activator 2
JAK2: Janus kinase 2
LPS: Lipopolysaccharide
lncRNA: Long noncoding RNA
MAPK: Mitogen-activated protein kinase
MCT1: Monocarboxylate transporter 1
miRNA: MicroRNA
mTOR: Mammalianarget of rapamycin
mmdA: Methylmalonyl-CoA decarboxylase
MYD88: Myeloid differentiation primary response 88
NAFLD: Non-alcoholic fatty liver disease
NF-κB: Nuclear factor-κB
NLRP3: NLR family pyrin domain containing 3
PD: Parkinson’s disease
PI3K: Phosphoinositide 3-kinase
PKM2: Pyruvate kinase isozyme 2
PPAR-γ2: Peroxisome proliferator-activated receptor-γ2
SARS-CoV-2: Severe acute respiratory syndrome coronavirus 2
SCFAs: Short-chain fatty acids
SMCT1: Sodium-coupled monocarboxylate transporter 1
STAT3: Signal transducer and activator of transcription 3
TLR4: Toll-like receptors 4
TNF-α: Tumor necrosis factor-α
Treg: Regulatory T cells
T2D: Type 2 diabetes
ZO-1: Zonula occludens protein 1
